# Enablers and barriers to primary health care access for Indigenous adolescents: a systematic review and meta-aggregation of studies across Australia, Canada, New Zealand, and USA

**DOI:** 10.1186/s12913-024-10796-5

**Published:** 2024-04-30

**Authors:** Stephen Harfield, Tara Purcell, Eliza Schioldann, James Ward, Odette Pearson, Peter Azzopardi

**Affiliations:** 1 UQ Poche Centre for Indigenous Health, University of Queensland, St Lucia, Australia; 2 School of Public Health, University of Queensland, Herston, Australia; 3 Aboriginal Health Equity, South Australian Health and Medical Research Institute, Adelaide, Australia; 4 School of Public Health, The University of Adelaide, Adelaide, Australia; 5 Global Adolescent Health Group, Maternal Child and Adolescent Health Program, Burnet Institute, Melbourne, Australia; 6 Adelaide Medical School, The University of Adelaide, Adelaide, Australia; 7 Centre for Adolescent Health, Department of Paediatrics, University of Melbourne, Melbourne, Australia

**Keywords:** Primary health care, Indigenous peoples, Adolescents, Australia, New Zealand, Canada, United States of America

## Abstract

**Background:**

Indigenous adolescents access primary health care services at lower rates, despite their greater health needs and experience of disadvantage. This systematic review identifies the enablers and barriers to primary health care access for Indigenous adolescents to inform service and policy improvements.

**Methods:**

We systematically searched databases for publications reporting enablers or barriers to primary health care access for Indigenous adolescents from the perspective of adolescents, their parents and health care providers, and included studies focused on Indigenous adolescents aged 10–24 years from Australia, Canada, New Zealand, and United States of America. Results were analyzed against the WHO Global standards for quality health-care services for adolescents. An additional ninth standard was added which focused on cultural safety.

**Results:**

A total of 41 studies were included. More barriers were identified than enablers, and against the WHO Global standards most enablers and barriers related to supply factors – providers’ competencies, appropriate package of services, and cultural safety. Providers who built trust, respect, and relationships; appropriate package of service; and culturally safe environments and care were enablers to care reported by adolescents, and health care providers and parents. Embarrassment, shame, or fear; a lack of culturally appropriate services; and privacy and confidentiality were common barriers identified by both adolescent and health care providers and parents. Cultural safety was identified as a key issue among Indigenous adolescents. Enablers and barriers related to cultural safety included culturally appropriate services, culturally safe environment and care, traditional and cultural practices, cultural protocols, Indigenous health care providers, cultural training for health care providers, and colonization, intergenerational trauma, and racism. Nine recommendations were identified which aim to address the enablers and barriers associated with primary health care access for Indigenous adolescents.

**Conclusion:**

This review provides important evidence to inform how services, organizations and governments can create accessible primary health care services that specifically meet the needs of Indigenous adolescents. We identify nine recommendations for improving the accessibility of primary health care services for Indigenous adolescents.

**Supplementary Information:**

The online version contains supplementary material available at 10.1186/s12913-024-10796-5.

## Background

Adolescence is an important period of development across the life-course, the period between childhood and adulthood, which is now broadly considered to be between the ages of 10–24 years [1]. This period of life is defined by critical biological, neurocognitive, and social role transitions [1, 2] and a manifestation of this is the shift from childhood diseases and illness to those more prevalent in adolescents, sexual and reproductive health, mental health, injury, drug and alcohol misuse, obesity, and early onset of chronic disease [2, 3]. Adolescence is also a critical time for engagement with health care services, as it is the phase where adolescents shift from accessing care with parents to accessing care independently, and a time to not only to address emergent health needs of adolescents, but also to identify and address risks for future and intergenerational health.

To meet the health and wellbeing needs of adolescents requires access to quality health care for all adolescents, which is now recognised in global health policy [4–6]. Accessible and responsive primary health care services are essential to addressing the health care needs of adolescents, as well as optimising opportunities for health promotion and prevention. Yet we know in many settings, adolescents infrequently access health care and/or forego care when it is needed [7]. Barriers to health care experienced by adolescents include a lack of experience and knowledge in relation to health care access, a lack of privacy and confidentiality, a lack of suitable services, location of services, cost, stigma, cultural and community attitudes, legislation and other legal frameworks which impact access for adolescents [8–10].

In Australia, adolescents and especially adolescent males are less likely than any other population group to access primary health care services [11]. Data from Australia’s universal health care insurance scheme Medicare indicates that in 2018–19, males aged 10–14 years on average received 5.8 Medicare services per capita, the lowest number of services than any other population group, followed by males aged 15–19 and 20–24 years who received on averaged 6.2 Medicare services per capita [12]. A study by Slade et al. [13], found Australian males aged 16–24 years had the lowest service use for mental health problems compared to any other population group (13% vs 18% for males aged 75–85 year-second lowest group). Similarly, in Canada, New Zealand and United States of America (USA), adolescents are less likely to use primary health care services than any other age groups [14–19]. Within these settings, Indigenous adolescents appear to have particularly low access to services, despite their greater health needs compared to their non-Indigenous counterparts [20–25]. In Australia, in 2022, 223,877 Aboriginal and Torres Strait Islander Peoples Health Assessment (Medicare Item 715) were conducted, 15% of those Health Assessments were competed by Aboriginal and Torres Strait Islander adolescents aged 15–24 years [26]. This corresponds to 20% of Aboriginal and Torres Strait Islander young population aged 15–24 years having had a Health Assessment in 2022 [26]. Despite the intentions of the Health Assessments, which recognises the unique health and wellbeing needs of Aboriginal and Torres Strait Islander people and encourages engagement in comprehensive primary health care [27], adolescents were less likely to use primary health care services than any other Aboriginal and Torres Strait Islander population group [20, 26]. To date, there has not been a systematic analysis of the enablers and barriers to primary health care access among Indigenous adolescents.

Australia, Canada, New Zealand, and USA all have similar policy frameworks for their Indigenous populations, funding mechanisms, and Indigenous-specific health care services [28–34]. These services have evolved from an identified need to deliver health care to Indigenous communities that is culturally safe, often as a result of mainstream services failing to provide culturally safe care for Indigenous communities [28]. However, these services have traditionally been designed to target the health needs of mothers, babies and young children (where excess mortality has historically occurred) and adults (burden of chronic illness) [35]. Indigenous adolescents may also experience unique obstacles due to the ongoing effects of colonisation and their impact on the social determinants of health – dispossession of land and country, intergenerational trauma, child removal, racism, and inequity [36–38]. Furthermore, Indigenous adolescents comprise a large proportion of the Indigenous population, for example, in Australia, Indigenous adolescents aged 10–24 years make up 30% of Australia’s Indigenous population [39] and experience a large burden of preventable ill health [21].

This review aimed to identify the enablers and barriers experienced by Indigenous adolescents accessing primary health care services as described by Indigenous adolescents, their family members and health care providers, from Australia, Canada, New Zealand, and USA.

## Methods

### Search strategy and selection criteria

The review study protocol was registered with PROSPERO [CRD42021268266]. This review was reported according to the Preferred Reporting Items for Systematic Reviews and Meta-Analyses (PRISMA) [40]. Literature from Jan 1, 2002, to December 13, 2023, were compiled from MEDLINE (PubMed), EBSCOhost (CINAHL Complete; and PsycInfo), Embase, Scopus, Web of Science (Core Collection), Ovid (EMCARE), Informit (Health Collection; Humanities & Social Sciences Collection; Indigenous Collection; and Aboriginal and Torres Strait Islander Health Bibliography), PHCRIS, Circumpolar Health, Native Health, and HealthInfoNet databases. Key search terms were used (Indigenous, adolescent, primary health care, Australia, Canada, New Zealand and USA) (Additional file 1) to identify studies from Australia, Canada, New Zealand and USA that identified enablers and barriers experienced by Indigenous adolescents accessing primary health care, including those with a focus on antenatal care, mental health, sexual health, drug and alcohol substance abuse and rehabilitation, from the perspective of either the individual, their parents/guardians/community members or healthcare providers. We focus on studies from Australia, Canada, New Zealand and USA, as Indigenous populations from these high-income countries have similar shared experiences of colonization and similar health policies [41, 42]. Indigenous peoples were defined as individuals and communities who identify as being Indigenous or First Nations [43]; and adolescents were defined as individuals between the ages of 10–24 years, as its algins with ‘contemporary patterns of adolescent growth and popular understandings of this life phase’ ([1], p.1). Studies that contained both Indigenous and non-Indigenous adolescents were included if greater than 50% of participants were Indigenous, and studies where the proportion of adolescents was representative of the adolescent population in the region studied [19], or data were disaggregated by Indigenous status. Studies were included if they were primary qualitative, quantitative, or mixed method studies, including evaluations, and reports and other grey literature (from PHCRIS, Circumpolar Health, Native Health, and HealthInfoNet databases). The review excluded review articles, editorials, and perspectives. Articles were limited to those published in English. Studies were excluded if they did not identify enablers and barriers experienced by Indigenous adolescents accessing primary health care or focused on preventative health intervention programs delivered outside of primary health care, including vaccination, contraception, and physical activity programs, screening programs (mental health, sexual health, cardiovascular health) and harm reduction initiatives (smoking cessation, needle, and syringe exchange), as we focused on the responsive care side of primary health care, e.g. treatment and management of acute and chronic conditions. The reference list of included studies were reviewed for additional studies.

The review team consisted of Aboriginal (SH, TP) and non-Indigenous individuals (PA, ES), with expertise in Aboriginal and Torres Strait Islander health (SH, TP, PA), adolescent health (PA), health services research (SH, PA) and systematic reviews (SH). Title and abstract and full-text review were conducted according to the inclusion and exclusion criteria by two independent reviewers (SH, TP, PA). Conflict between reviewers were resolved by reaching an agreement through discussion and or with another reviewer. Inter-rater reliability between reviewers at title and abstract screening and full text review were moderate (0.67 and 0.73, respectively).

### Data extraction and quality appraisal

Data extraction was completed by two independent reviewers (SH and ES). Information extracted from each study included, but was not limited to, author, year, country where study was conducted, geographical setting, population and characteristics of participants, study design, phenomena of interest, and identified enablers and barriers experienced by Indigenous adolescents accessing primary health care. Due to the small number of quantitative studies, quantitative data was converted into ‘qualitized data’. This involved transforming quantitative data into textual descriptions or narrative interpretation of the quantitative results [44]. Following the data extraction process, quality appraisal was performed by two independent reviewers (SH, ES) using tools developed by Joanna Briggs Institute [44]; and the Aboriginal and Torres Strait Islander Quality Appraisal Tool, which assesses the quality of health research from an Indigenous perspective [45]. The Aboriginal and Torres Strait Islander Quality Appraisal Tool was modified to be inclusive of Indigenous populations from Australia, Canada, New Zealand, and USA. The abstract, full-text review, data extraction and quality appraisal were completed using EndNote and Covidence Software [46, 47].

### Data analysis

The World Health Organization (WHO) *Global standards for quality health-care services for adolescents* [6] (Global standards) were adapted and used to explore the enablers and barriers to primary health care for Indigenous adolescents. The Global standards provide guidance for improving the quality of health care services and delivery for adolescents and consider how health systems respond to the health needs of adolescents using a human rights-based approaches to health. In addition to the eight Global standards [6], we included a ninth standard: cultural safety, which encapsulates the unique cultural needs and experience of Indigenous adolescents when accessing primary health care services (Table 1). Cultural safety is defined as:

**Table 1:** Modified Global standards for quality health-care services for adolescents [6]

Key concepts	Standards
Adolescents’ health literacy (demand)	Standard 1. The health facility implements systems to ensure that adolescents are knowledgeable about their own health, and they know where and when to obtain health services.
Community support (demand)	Standard 2. The health facility implements systems to ensure that parents, guardians and other community members and community organizations recognize the value of providing health services to adolescents and support such provision and the utilization of services by adolescents.
Appropriate package of services (supply)	Standard 3. The health facility provides a package of information, counselling, diagnostic, treatment and care services that fulfils the needs of all adolescents. Services are provided in the facility and through referral linkages and outreach.
Providers’ competencies (supply)	Standard 4. Health-care providers demonstrate the technical competence required to provide effective health services to adolescents. Both healthcare providers and support staff respect, protect and fulfil adolescents’ rights to information, privacy, confidentiality, non-discrimination, non-judgemental attitude, and respect.
Facility characteristics (supply)	Standard 5. The health facility has convenient operating hours, a welcoming and clean environment and maintains privacy and confidentiality. It has the equipment, medicines, supplies and technology needed to ensure effective service provision to adolescents.
Equity and non-discrimination (supply)	Standard 6. The health facility provides quality services to all adolescents irrespective of their ability to pay, age, sex, marital status, education level, ethnic origin, sexual orientation, or other characteristics.
Data and quality improvement (demand)	Standard 7. The health facility collects, analyses, and uses data on service utilization and quality of care, disaggregated by age and sex, to support quality improvement. Health facility staff is supported to participate in continuous quality improvement.
Adolescents’ participation (demand)	Standard 8. Adolescents are involved in the planning, monitoring and evaluation of health services and in decisions regarding their own care, as well as in certain appropriate aspects of service provision.
Cultural safety (supply)	Standard 9. Adolescents experience culturally safe care, which reflects their own culture and practices, including language, traditional healing and medicine, cultural protocols; the presence and involvement of Indigenous health care providers in the delivery of care; and acknowledgement of the historical context of colonisation and racism and their correlation with health and wellbeing and the delivery of care.

Cultural safety is not defined by the health professional, but is defined by the health consumer’s experience—the individual’s experience of care they are given, ability to access services and to raise concerns. The essential features of cultural safety are:

- *An understanding of one’s culture*
- *An acknowledgment of difference, and a requirement that caregivers are actively mindful and respectful of difference(s)*
- *It is informed by the theory of power relations; any attempt to depoliticise cultural safety is to miss the point*
- *An appreciation of the historical context of colonisation, the practices of racism at individual and institutional levels, and their impact on First Nations people’s living and wellbeing, both in the present and past*
- *Its presence or absence is determined by the experience of the recipient of care and not defined by the caregiver * [48]

Furthermore, these standards were divided into two groups: supply (individual or community level factors that facilitate or prevent access to health care services) and demand (health care system level factors that facilitate or prevent health care service uptake), as health care access is often considered in relation to these two concepts [49]. Findings were thematically analyzed and synthesized using meta-aggregation [44] under each Global standard, and analyzed by service type/focus (primary health care, mental health, and sexual and reproductive health), and urban status, as most Indigenous health research is conducting in rural and remote settings [50]. Urban was defined as the area or region surrounding a (capital) city [51].

## Results

**Fig. 1:**
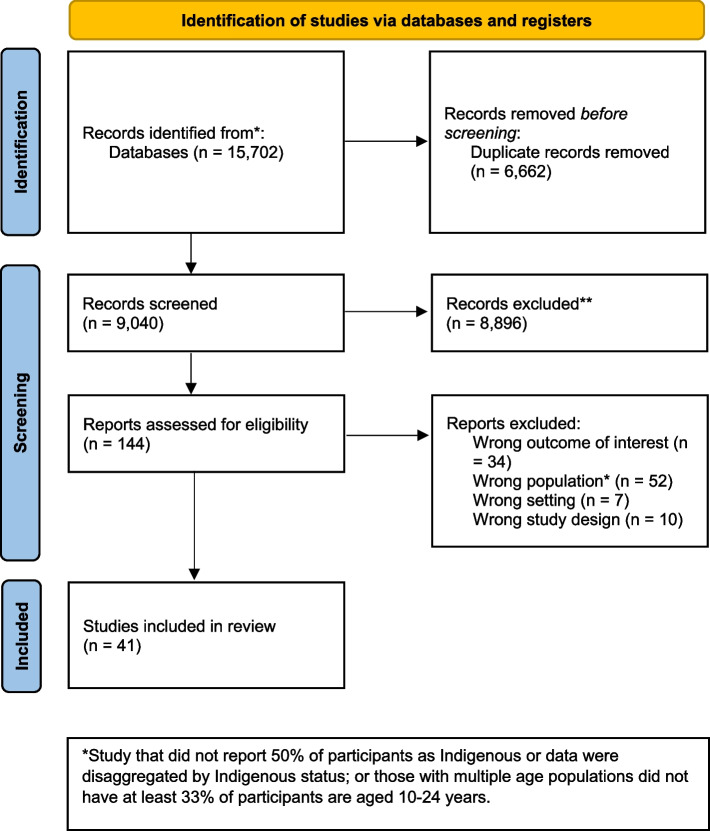
PRISMA 2020 flow diagram

Our search identified 15,702 articles. After abstract review (screening), 144 were deemed potentially relevant. Of these, 103 were excluded after full text review (Additional file 1). In total 41 studies were included in the systematic review (Fig. 1).

Of the 41 studies, most had an overall high-quality assessment against the Joanna Briggs Institute tools except for two studies (Additional file 1) and inter-rater reliability between the two reviewers was high [52, 53]. However, against the Aboriginal and Torres Strait Islander Quality Appraisal Tool, which assesses research quality from an Indigenous perspective, 12 rated high [54–64], 12 medium [61, 65–74], and 17 poor [24, 52, 53, 75–86] (Additional file 1). The overall quality was limited by insufficient description of most criteria, particularly on agreements in relation to intellectual and cultural property, control over the collection and management of research material, capacity strengthening and opportunities to learn, Indigenous research paradigm, and Indigenous governance.

Table 2 summarizes the characteristics of included studies. The majority of studies were from Australia [52, 53, 55, 56, 59, 61–63, 69, 74, 76, 78–82, 85, 87, 88], followed by Canada [54, 57, 58, 64, 66, 68, 86, 89], New Zealand [60, 70–73, 75, 90, 91], and USA [24, 65, 67, 77, 83, 84]. Of the 41 studies, 20 were conducted in or focused on primary health care services [52, 55, 56, 59, 61, 65, 66, 68–70, 72, 79–81, 83, 85, 87–90], 15 were conducted on mental health [53, 54, 57–60, 63, 67, 73, 74, 77, 80, 82, 84, 85], ten on sexual and reproductive health including maternal health [55, 61, 62, 66, 70, 71, 79, 83, 87, 91], and the remaining studies were on health care access [56, 68, 72, 81], health and wellbeing [24, 86], alcohol and other drugs [75, 76], transgender and two-spirit health [65], asthma [64], skin infections [69], physical disability [78], and health knowledge mobilization [89]. Most studies reported results from the perspective of adolescents [24, 52–56, 60–64, 66, 67, 70, 71, 73, 75–79, 81–84, 87, 90, 91], with the majority of studies involving older adolescents, and conducted in non-urban or a combination of non-urban and urban settings [24, 52–66, 68, 69, 72–75, 77–81, 87–89, 91]. Results are presented based on settings and themes – primary health care, mental health, sexual and reproductive health, and urban status.

**Table 2:** Characteristics of included studies

**Author (Year)**	**Country**	**Urban status **	**Population**	**Study setting**	**Area of health **
Ameratunga et al (2019) [75]	New Zealand	Urban and non-urban	Non-Indigenous and Indigenous adolescents (male and female, aged 12-19 years)	High schools (national)	Alcohol and other drugs
Angelino et al (2020) [65]	USA	Urban and non-urban	Health care providers	Indigenous primary health care service and hospital	Transgender and two-spirit health care
Auger (2019) [54]	Canada	Urban and non-urban	Indigenous adolescents (male and female, aged 19-24 years) and adults	Community-based	Mental health and wellbeing
Bell et al (2020) [55]	Australia	Non-urban	Indigenous adolescents (male and female, aged 16-21 years)	Community-based: Indigenous primary health care service	Sexual health
Canuto et al (2018) [56]	Australia	Urban and non-urban	Indigenous adolescents (male, aged 19-24) and adults	Community-based	Primary health care and access
Corosky et al (2016) [66]	Canada	Non-urban	Indigenous adolescents (male and female, aged 16-22 years) and community leaders	Community-based: High school, and Indigenous primary health care service	Sexual and reproductive health (primary health care)
Dickerson et al (2011) [67]	USA	Urban	Indigenous adolescents (male and female, aged 14-17 years), parents and service providers	Community-based	Mental health and alcohol and other drugs
Dowsett et al (2019) [76]	Australia	Urban	Indigenous adolescents (male and female, aged 16–24 years), and adults	Community-based: youth and alcohol and other drug services	Alcohol and other drugs
Etter et al (2019) [57]	Canada	Non-urban	Health care providers	Indigenous Community mental health service	Mental health
Fraser et al (2021) [68]	Canada	Non-urban	Health and social services providers	Indigenous primary health care services and social services	Primary health care and other health services and social services
Freedenthal et al (2007) [77]	USA	Urban and non-urban	Indigenous adolescents (male and female, aged 15-21 years)	Community-based	Mental health
Garrett et al (2022) [90]	New Zealand	Urban	Non-Indigenous and Indigenous adolescents (aged 15-to 25-year olds)	Cross-sectional online survey	Primary health care and telehealth
Greenstein et al (2016) [78]	Australia	Non-urban	Parents and carers, and Indigenous children and adolescents (male and female, aged 12 to 21 years)	Community-based physiotherapy service	Physical disability
Hendrickx et al (2020) [69]	Australia	Non-urban	Parents/carers, health care providers and other service providers	Indigenous and non-Indigenous primary health care services	Skin infections
Hummel et al (2022) [89]	Canada	No-urban	Parents and caregivers of Indigenous children (aged ≤18-year-olds)	Indigenous health service	Health knowledge mobilisation
Hutt-MacLeod et al (2019) [58]	Canada	Non-urban	Health care providers	Indigenous community mental health service	Mental health
Johnston et al (2015) [79]	Australia	Non-urban	Health care providers and non-Indigenous and Indigenous adolescents (aged 15-24 years)	Indigenous and non-Indigenous primary health care services, sexual health clinics, school-based health, and youth services	Sexual and reproductive health
Kalucy et al (2019) [59]	Australia	Urban and non-urban	Health care providers	Indigenous primary health care service	Mental health
Kurtin et al (2009) [80]	Australia	Non-urban	Health care providers	Primary health care services and community mental health services	Mental health
Lau et al (2012) [24]	USA	Urban and non-urban	Non-Indigenous and Indigenous adolescents (aged 10-17 years)	Community-based	health and wellbeing
Lawton et al (2016) [70]	New Zealand	Urban	Indigenous adolescents (females aged 14 to 19)	Antenatal, primary health care and youth services, and educational providers	Sexual and reproductive health
Makowharemahihi et al (2014) [71]	New Zealand	Urban	Indigenous adolescents (female, aged 14-20 years	Health, education, and social service services	Maternal and reproductive health
Martel et al (2020) [72]	New Zealand	Non-urban	Health care providers	primary health care services	Adolescent health
McClintock et al (2013) [73]	New Zealand	Urban and non-urban	Indigenous adolescents	Child and adolescent mental health services	Mental health
McClintock et al (2016) [60]	New Zealand	Urban and non-urban	Indigenous adolescents (aged 12-19 years)	Child and adolescent mental health services	Mental health
Mooney-Somers et al (2009) [61, 87]	Australia	Non-urban	Indigenous adolescents (male and female, aged 17-24 years), adults	Indigenous and non-Indigenous primary health care services, and sexual health clinics	Sexual health
Mooney-Somers et al (2009) [61, 87]	Australia	Non-urban	Indigenous adolescents (male and female, aged 17-24 years), adults	Indigenous and non-Indigenous primary health care services, and sexual health clinics	Sexual health
Reibel et al (2015) [62]	Australia	Urban and non-urban	Indigenous adolescents (females, aged 16–21 years), community members and health care providers	Community-based	Maternal and reproductive health care
Robards et al (2019) [81]	Australia	Urban and non-urban	Non-Indigenous and Indigenous adolescents (all genders, aged 12–24 years)	Community-based	Health care access, including primary health care
Rose et al (2021) [91]	New Zealand	Urban and non-urban	Non-Indigenous and Indigenous adolescents (aged 15-to 24-year-olds)	Online survey	Sexual health and access to health care
Sabbioni et al (2018) [82]	Australia	Urban	Indigenous adolescents	Community mental health service	Mental health
Saftner et al (2014) [83]	USA	Urban	Indigenous adolescent (females, aged 15-19 year)	Indigenous primary health care services	Health care access, including primary health care; sexual health
Salvador et al (2016) [84]	USA	Urban	Indigenous adolescents (male and female, aged 11-19 years), parents and education providers	School-based	Mental health
Santhanam et al (2006) [74]	Australia	Non-urban	Health care providers	Child and Youth Mental Health services	Mental health
Schultz et al (2019) [63]	Australia	Non-urban	Indigenous adolescents (male and female, aged 15 to 24 years), adults	Community-based	Mental health
Stewart et al (2013) [64]	Canada	Urban and non-urban	Indigenous children and adolescents (male and female aged, 12 to 19 years), and parents and carers	Community-based	Respiratory - asthma
Warwick et al (2019) [52]	Australia	Non-urban	Indigenous adolescents (aged 16 to 25-years-old)	Indigenous primary health care service	Primary health care access
Warwick et al (2021) [88]	Australia	Non-urban	Health care providers	Indigenous health service	Primary health care access
Westerman (2010) [53]	Australia	Urban and non-urban	Indigenous adolescents (aged 13–17 years)	Mental health services	Mental health
Williamson et al (2010) [85]	Australia	Urban	Parents and health care providers	Indigenous primary health care services	Mental health
Yi et al (2015) [86]	Canada	Urban	Health care providers and other providers	Community-based	Health and wellbeing

### Primary health care

Table 3 describes the enablers and barriers associated with Indigenous adolescents accessing primary health care services from the perspective of adolescents and health care providers and parents/guardians/community members. Of the 20 studies conducted in or focused on primary health care services [52, 55, 56, 59, 61, 65, 66, 68–70, 72, 79–81, 83, 85, 87–90], 11 of those studies were from the perspective of adolescents [52, 55, 56, 61, 66, 70, 79, 81, 83, 87, 90], with most from the perspective of female and older adolescents, and four were conducted in urban settings [70, 83, 85, 89, 90] with another four conducted in both urban and non-urban settings [56, 59, 65, 81]. Both adolescents and health care providers and parents/guardians/community members reported more barriers than enablers, with more enablers and barriers identified under the five standards relating to supply by both groups. No study reported enablers and barriers associated with the data and quality improvement standard (demand).

**Table 3:** Enablers and barriers associated with Indigenous adolescents accessing primary health care services from the perspective of adolescents and health care providers and parents/guardians/community members

		**Enablers**		**Barriers**	
		**Adolescents**	**Health care providers and parents/guardians/community members**	**Adolescents**	**Health care providers and parents/**[89]**guardians/community members**
**Supply**	**Appropriate package of services**	**• Care that meets the needs of adolescents** [52, 83] **• Provision of additional services**: outreach services [52, 55], telehealth [90], transport [52, 61, 83, 87], interpreter [52] and social support such as ‘Women’s nights’ [55], and health hardware [87] facilitated access to services **• Flexible approach to care and pathways** facilitated by providers which ensured care was received [70] **• Availability of gender-matched health care providers** [52] **• Social media to promote services** and increase engagement [56]	**• Adolescent appropriate care** that centres [69, 88] and is designed for adolescents [72] **• Provision of additional services**: transport [59] **• Flexible approach to care and pathways** facilitated and supported by providers which ensured care was received [59, 88] **•** Technology and **adolescent specific tools/assessments** assist with the health and wellbeing assessment of adolescents [72] **• Engagement staff** to engage with and follow up adolescents [88]	**• Services not available when required** [55, 70, 87], including a lack of transport [52] hindered access **• Not receiving care that is needed** [90] **• Waiting times** [52] **•** The **disconnect between health services** [70] impacted continuity of care **• Limited access to health hardware**, such as contraception including condoms [61, 70, 87] led to adolescents taking risks **• Workforce challenges**: appropriately aged and gendered providers [83], and the transient nature of the providers in remote communities [52, 66] reduced access and continuity of care	**• Services not available when required** [85], including specialist services at Indigenous health services [59] or adolescent specialist [59] **• Wait times** [88] **• A lack of follow up and recall** makes it difficult for staff to provide care [88] **• A lack of staffing and resources** [88], **including within community** to provide services and care needed, e.g., emergency housing, in-community alcohol and drug rehabilitation services, psychotherapy, and financial assistance [68] **•** The **disconnect between health services** – mainstream and specialist services [59] and the division of child-adolescent and young adult services [81] impacted continuity of care **• Workforce challenges**: a lack of providers [65] **•** The **absence of appropriate adolescent tools, guidelines, and established pathway** [59] impeded assessment and care
	**Providers’ competencies**	**• Providers who built trust, respect, and relationships with adolescents and families** were able to facilitate health seeking behaviour, health service engagement and access to services [52, 55, 61, 66, 83, 87] **•** Providers who maintain **privacy and confidentiality** [52, 83] and were honest, non-judgemental and had good communication skills [52, 83] were seen to have **positive provider qualities**, which provided for a **positive experience** [61, 87]	**• Providers who built trust, respect, and relationships with adolescents and families** were able to facilitate health seeking behaviour, health service engagement and access to services [59, 65, 69, 88] **•** Providers receive **education and training** that includes Indigenous culture, traditional healing, and gender-affirming care [65] **• Specialist adolescent providers** have the ability to provide care that meets the health and wellbeing needs of adolescents [72] **•** Providers who were welcoming, attentive and non-judgemental, had good communication skills, positive attitude [59, 69, 79, 88], and open door policy [88] and went beyond the usual level of care [81, 88] were seen to have **positive provider qualities** and **maintained confidentiality** [88]	**• A lack of provider privacy, confidentiality, and trust** [52, 66] hindered adolescents’ access to health services **• Previous negative experience with providers** [61, 83, 87] often meant adolescents and their families were less likely to utilise services	**• A lack of provider privacy, confidentiality, and trust** [59, 79] **• Previous negative experience with providers** [69] often meant adolescents and their families were less likely to utilise services **• Providers reluctant to engage with adolescents and their families** [68] **• A lack of specific training and experience** in adolescent health [59], Indigenous cultural [65, 69], LGBTQ [65] or other training requirements [69] affected providers ability to provider appropriate care **• Provider isolation** is a challenge for those working in remote communities [69]
	**Facility characteristics**	**• Services with same day appointments, walk-ins** [83], and being able to book appointment [52], including using an online appointment systems [81] facilitate access **• Welcoming and safe spaces** [52, 55, 81] made services more inviting	**• Welcoming and safe spaces** [65, 69] made services more inviting	**• Location of services** [55], transport including proximity to public transport [61, 87], and inability to pre-book appointments [55] limited accessibility **•** A lack of separate entrances and waiting rooms for men and women meant **facilities were not culturally appropriate**, and **privacy of waiting rooms** was also an issue [52, 55]	**• Location of services** [65, 79], transport including proximity to public transport [59, 65, 79], visibility of service [79], opening hours [79] limited accessibility **• A lack of privacy in waiting areas** [88]
	**Equity and non-discrimination**	**• Low or no cost services** [56, 83, 90] facilitate access	**• Low or not cost services** [69] facilitate access	**• Cost of services** [70] and **adolescents’ personal circumstances** [61] often prevent or limited service access **•** The **experiences of discrimination** often lead to adolescents foregoing care [81] **• Embarrassment, shame, or fear** [52, 55, 61, 66, 81, 87] often prevent adolescents from seeking and accessing services	**• Cost of services** [79] and **adolescents personal circumstances** [65] often prevent or limited access to service **• Embarrassment, shame, or fear** [69, 79, 80] often prevent adolescents from seeking and accessing services **•** The **outdated medical systems** and the way it incorporates or excludes, into gender-affirming care, and use of terminology and understanding of LGBTQ, transgender or Two-Spirit often perpetuate stereotypes and discrimination [65]
	**Cultural safety**	**•** Adolescents sought out **Indigenous specific services** [61, 83] or services that provided **culturally safe environments and care**, which reflected adolescents’ culture and beliefs [55, 61, 83, 87] **•** The presence of **Indigenous health care providers and their role in the provision of culturally safe care** [52] **• Cultural protocols** were observed with providers and adolescents being the same gender [52, 55]	**•** Adolescents sought out services that provided **culturally safe environments and care**, which reflected adolescents’ culture and beliefs [72] and facilitated accessibility [69] **•** The presence of **Indigenous health care providers and their role in the provision of culturally safe care**, as cultural brokers were essential to adolescents receiving culturally appropriate care [85]	**• Racism** [81] impacts adolescents and their family’s ability to engage with and access services **• A lack of Indigenous health care providers** [52]	**• Colonisation and intergenerational trauma** impacts adolescents and their family’s ability to engage with and access services [65] **•** Acceptability of mainstream services to provide culturally safe care, **not all services are culturally safe** for Indigenous adolescents [59, 65] **• Absence of culture and language** were barriers to engaging and communicating with adolescents and their families [69] **• No integration or disconnect between western and traditional health & cultural practices** [69, 80] **• Absence of cultural protocols**: restrictions around family relationships, skin groups and gender [69] **• A lack of Indigenous health care providers** [69] **•** Many **providers had inadequate training:** cultural [59] and had limited or no understanding of Two-Spirit and gender and sexuality within the Indigenous context [65] **• Medical system which perpetuates colonial norms** such as inequality and marginalisation [65]
**Demand**	**Adolescent health literacy**	**• Adolescents’ willingness to seek health care** [61, 87] and awareness of services [56] enable them to navigating the health care system **• Adolescents support other adolescents** by providing advice and reassurance to reduce shame in relation to seeking help [87]	**• Delivery of health promotion in adolescent settings**, e.g., in schools, and incentives [88]	**• A lower level of health literacy**, including a lack of health seeking behaviours and awareness of services [52, 55, 56, 61, 81], communication skills [55], adolescents’ attitude [55], prioritising their own health [52] were barriers to adolescents seeking care and navigating the health care system **• Adolescents’ ability to make and maintain appointments** [61, 87]	**• A lower level of health literacy** [69, 88], including awareness of services [79], health seeking behaviour [65], and perceived need for help [69] or prioritising their own health [52] were barriers to adolescents seeking care and navigating the health care system **• Adolescents’ ability to maintain appointments** [59]
	**Community support**		**• Parent/family and community engagement and involvement in care** was essential to adolescents receiving care [69]	**A lack of community and family support** [55] hindered adolescents’ access to care	**• A lack of guidance on how to communicate with adolescents** about safe sex, mental health, and suicide [89] **• A lack of community and family acceptance or support** [65] and involvement in treatment decisions [69] hindered adolescents’ access to care **• Family issues** [59] competing priorities of parents [69] and reliance on family [59] impeded adolescents receiving care **• Fear of child welfare services and police involvement** [69, 85] deterred families seeking care for their adolescents
	**Data and quality improvement**				
	**Adolescents’ participation**				**• Disempowerment** especially among females and LGBTQI adolescents [66]

Factors identified as Providers’ Competencies were common enablers reported by adolescents, including providers who built trust, respect, and relationships with adolescents and families, these were described as facilitating health seeking behavior, health service engagement and access to services [52, 55, 61, 66, 83, 87]; and providers who maintain privacy and confidentiality [52, 83] and were honest, non-judgmental and had good communication skills were seen to have positive provider qualities, which provided for a positive experience [52, 61, 83, 87]. As were factors identified relating to Appropriate Package of Services, particularly the provision of additional services such as outreach, telehealth, social support, and transport facilitated access to services among adolescents [52, 55, 61, 83, 87, 90]. Adolescents also reported factors related to Cultural Safety, specifically the provision of culturally safe environments and care, which acknowledged and reflected adolescents’ culture and beliefs through the inclusion of cultural practices and protocols, these were also considered enablers by adolescents [52, 55, 61, 83, 87]. Embarrassment, shame, or fear (Equity and Non-discrimination) was a common barrier reported by adolescents and often prevent adolescents from seeking and accessing services [55, 61, 66, 81, 87]. Similarly, a low level of health literacy among adolescents including a lack of health seeking behaviors and awareness of services were barriers to adolescents seeking care (Adolescent Health Literacy) [55, 56, 61, 81].

Health care providers and parents/guardians/community members also reported, providers who built trust, respect, and relationships with adolescents and their families (Providers’ Competencies) [59, 65, 69, 88], and positive provider qualities, such as providers who were welcoming, attentive and non-judgmental, had good communication skills, and a positive attitude [59, 69, 79, 88], were commonly reported enablers associated with Indigenous adolescents accessing primary health care services. Health care providers and parents/guardians/community members identified several factors related to Cultural Safety, which were barriers to adolescents and their family’s ability to engage with and access services: colonization and intergenerational trauma limited understanding and knowledge of Indigenous culture and systemic issues such as racism, marginalization, and inequality impact access [65]; not all services were considered culturally safe for Indigenous adolescents [59, 65]; the absence of culture and language were barriers to engaging and communicating with adolescents and their families [69]; no integration or disconnect between western and traditional health and cultural practices [69, 80]; a lack of Indigenous health care providers [69]; and inadequate training on Indigenous cultural [59] and gender and sexuality within the Indigenous context [65]. Similarly, health care providers and parents/guardians/community members recognized several factors as barriers associated with Providers’ Competencies, such as a lack of provider privacy, confidentiality, and trust [59, 79]; previous negative experience with providers [69]; providers reluctant to engage with adolescents and their families [68]; and a lack of specific training and experience in adolescent health, Indigenous culture, LGBTQ [59, 65, 69], impacted adolescents and their families utilization of services and affected providers ability to provider appropriate care.

### Mental health

Of the fifteen studies conducted in or focused on mental health services, adolescents and health care providers and parents/guardians/community members reported more enablers than barriers (Table 4). Adolescents reported self-determination and empowerment (Adolescents’ Participation), and their involvement in care and decision making to be important enablers related to adolescents’ participation [60, 67, 73, 82]. This was also recognized by health care providers and parents/guardians/community members as an enabler [67]. Additionally, adolescent, reported several factors associated with Appropriate Package of Services, such as availability of providers and services, and flexible approach to care and pathways [60, 73, 82]; Providers’ Competencies [53, 60, 82]; and Cultural Safety, for example, the provision of traditional healing practices, and the Indigenous workforce and their role in the provision of cultural safe care [53, 60, 67, 82], as significant enablers to their access and utilization of mental health services. Adolescents reported barriers across seven of the nine standards, except for Data and Quality Improvement, and Adolescents’ Participation.

**Table 4:** Enablers and barriers associated with Indigenous adolescents accessing primary and community-based mental health services from the perspective of adolescents and health care providers and parents/guardians/community members

		**Enablers**		**Barriers**	
		**Adolescents**	**Health care providers and parents/guardians/community members**	**Adolescents**	**Health care providers and parents/guardians/community members**
**Supply**	**Appropriate package of services**	**• Care is holistic** [82] **• Availability of providers and services** [60], including outreach services [53, 67], and transport [73] facilitated access **• Flexible approach to care and pathways** facilitated by providers which ensured care was received [73, 82] **• Continuity of care between providers and services**, including referrals [53] **• Funding for services and resources** [54]	**• Adolescent appropriate care** that centre adolescents, families, and community in the delivery of care [58] **• Availability of providers and services** [57, 74], and **reduced waiting times** [58] **• Provision of services** including multi-disciplinary team [58], outreach services [58, 67, 74], and transport [59, 67] facilitated access **• Flexible approach to care and pathways** facilitated by providers which ensured care was received [59] **• Continuity of care between providers and services**, including referrals [58] **• Funding for services and resources** [58] **• Social media to promote services** and increase engagement with adolescents [58]	**• Services not available when required** [61, 77, 84]	**• Services not available:** outreach services [67] and access to specialist services including adolescent specialist [58, 59] at Indigenous primary health care services [59] **•** The **disconnect between health services** – Indigenous, mainstream and specialist services [59], and the division of child-adolescent and young adult services [57] impacted continuity of care **•** The **absence of appropriate adolescent tools, guidelines, and established pathway** [59] impeded assessment and care **• A lack of sustainable funding and resources** [85] affected service provision
	**Providers’ competencies**	**• Providers who engaged in the community** [53], **and built trust, respect, and relationships with families and adolescents** [73, 84] were able to facilitate health seeking behaviour, health service engagement and access to care **•** Providers who maintain **privacy and confidentiality** [84], were able to communicate with adolescents [73], and had **positive provider qualities** [84] were more likely to engage with adolescents **•** Providers received **education and training**: Indigenous culture [73, 82], were able to acknowledge family’s spiritual and cultural beliefs [60]	**• Providers who engaged in the community** [58], **and built trust, respect, and relationships with families and adolescents** [57–59, 67] were able to facilitate health seeking behaviour, health service engagement and access to care **• Positive provider qualities** [58, 59] were more likely to engage with adolescents **•** Providers received **education and training**: mental health [57]	**• A lack of provider privacy and confidentiality** [84] hindered adolescents’ health service access **• Previous negative experiences with providers** impacted adolescents’ future health seeking behaviour [53] **• A lack of specific training and experience** Indigenous cultural [54], mental health [54]	**• A lack of provider privacy and confidentiality** [59] and **trust in providers** [64] hindered adolescents’ health service access **• A lack of specific training and experience** in adolescent health [59] and Indigenous culture [59]
	**Facility characteristics**	**• Location of service** [53, 73] **and opening hours** [73] made services more accessible to adolescents **• Welcoming and safe space** [84] made services more inviting	**• Services opening hours** [58] made services more accessible to adolescents **• Welcoming and safe space** [58] made services more inviting	**• Transport** [63] is a barrier to adolescents accessing services	**• Location of services** including proximity to public **transport** [59]
	**Equity and non-discrimination**			**• Cost of services** [64, 77] **• Embarrassment, shame, or fear** [53] often prevent adolescents from seeking and accessing services	**• Cost of services** [64] **• Embarrassment, shame, or fear** [57, 80] often prevent adolescents from seeking and accessing services
	**Cultural safety**	**•** Adolescents sought out services that provided **culturally safe environment and care** [53, 54, 60] and the inclusion and centring of culture [73, 82] **• Provision of traditional health practices** [53, 60] and cultural activities [67] **• Indigenous workforce and their role in the provision of culturally safe care** [53, 82]	**•** Adolescents sought out services that provided **culturally safe environment and care** [74] **• Provision of traditional health practices** [57, 67] and cultural activities [67] **• Indigenous workforce and their role in the provision of culturally safe care** [57, 59, 74, 85]	**• A lack of culturally appropriate services** [53, 84] **• Absence of cultural protocols:** gender differences between adolescents and providers [53] **• A lack of Indigenous health care providers** [53]	**• A lack of culturally appropriate services** [59, 64, 67] including access to Indigenous specific health services [67] **• No integration or disconnect between traditional and cultural practices** [67, 80] **• A lack of Indigenous health care providers** [67]
**Demand**	**Adolescent health literacy**		**• Health literacy** [57, 58] **and health seeking behaviour** [57] including awareness of services [58, 67] enabled adolescents to seek care and navigate the health care system	**• Adolescent perceived need for help** [77] prevented them from seeking care **• Adolescents lacked independence in relation to health seeking** [84]	**• Adolescents’ ability to make and maintain appointments** [59]
	**Community support**	**• Parent/family and community engagement and involvement in care** [67, 73, 82]	**• Health literacy and health seeking behaviours of families** [57, 58] **• Parent/family and community engagement and involvement in care** [57, 58, 67, 74]	**• A lack of community engagement and involvement** in services and programs [54]	**• A lower level of health literacy of parents and families** [57] hindered adolescents’ access to care **• A lack of community engagement and involvement** in services and programs [54, 57, 58] **• Family issues and reliance on family** [59] impeded adolescents’ access to care **• Fear of child welfare services** and police involvement [85]
	**Data and quality improvement**	**•** Adolescents desired an **opportunity to provide feedback on the quality of service** [73] **• Evaluation of cultural competencies** within service delivery [53]			
	**Adolescents’ participation**	**• Self-determination and empowerment of adolescents** [73, 82] and their **involvement in care and decision making** [60, 67, 73, 82]	**• Adolescent engagement with services** through cultural and other activities [58] and their **involvement in care and decision making** [67]		**• No adolescent engagement in care and services** [57]

Health care providers and parents/guardians/community members reported several enablers which were identified relating to Appropriate Package of Services: adolescent appropriate care [58]; availability of providers and services [57, 74]; provision of services including multi-disciplinary team, outreach services and transport [58, 59, 67, 74]; flexible approach to care and pathways [59]; continuity of care between providers and services [58]; funding for services and resources [58]; and the use of social media to promote services and increase engagement with adolescents [58], were all considered enablers that facilitated access to care for adolescents. Additionally, health care providers and parents/guardians/community members reported several enablers relating to all other standards expect for Equity and Non-discrimination, and Data and Quality Improvement (Table 4). Similarly, health care providers and parents/guardians/community members reported barriers across most standards except for Data and Quality Improvement.

### Sexual and reproductive health

Of the ten studies conducted in or focused on sexual and reproductive health, a similar number of enablers and barriers were identified, with most of them associated with the five standards related to supply and reported by adolescents (Table 5). Most of the enablers reported by adolescents related to Appropriate Package of Services, Providers’ Competencies, and Cultural Safety, whereas the majority of the barriers related to Appropriate Package of Services, Providers’ Competencies, Facility Characteristics, and Adolescent Health Literacy. Barriers identified as Facility Characteristics included, location of services [55] and access to and proximity to public transport [61, 87], opening hours [79], inability to pre-book appointments [55], and a lack of separate entrances and waiting rooms for men and women and privacy of waiting rooms [55]; and barriers associated with Adolescent Health Literacy include, an assumed or lower level of health literacy [55, 61, 91], communication skills and attitudes of adolescents, and adolescents’ ability to make and maintain appointments [61, 87].

**Table 5:** Enablers and barriers associated with Indigenous adolescents accessing primary and community-based sexual and reproductive health services from the perspective of adolescents and health care providers and parents/guardians/community members

		**Enablers**		**Barriers**	
		**Adolescents**	**Health care providers and parents/guardians/community members**	**Adolescents**	**Health care providers and parents/guardians/community members**
**Supply**	**Appropriate package of services**	**• Adolescent appropriate care** that meets the needs of adolescents [83] **• Provision of additional services:** outreach services [55, 91], transport [61, 83, 87], and social support such as ‘Women’s nights’ [55] and health hardware [87] **• Flexible approach to care and pathways** facilitated by providers which ensured care was received [70]	**• Availability of providers** [57]	**• Services not available when required** [55, 70, 87] including options of providers [71] **• Limited access to health hardware**, such as contraception including condoms [61, 70, 87] **•** The **disconnect between health services** including allied health [70] **• Workforce challenges:** appropriately aged and gendered providers [83] and the transient nature of the providers in remote communities [66]	**• Workforce challenges:** a lack of providers [65] the transient nature of the providers in remote communities [66]
	**Providers’ competencies**	**• Providers who built trust, respect, and relationships with adolescents and families** [55, 61, 62, 66, 71, 83, 87] **•** Providers who maintain **privacy and confidentiality** [83, 91] were seen to have **positive provider qualities** [79, 83] and went beyond the usual level of care [71], which provided for a **positive experience** [61, 87] **• Reassuring adolescent** and normalising conversations about sexual health [91]		**• A lack of provider privacy, confidentiality, and trust** [62, 66, 91] **• Previous negative experience with providers** [61, 71, 83, 87] **• A lack of provider communication skills** [71] often meant adolescents and their families were less likely to utilise services	**• A lack of provider privacy, confidentiality, and trust** [62, 66, 79]
	**Facility characteristics**	**• Services with same day appointments and walk-ins** [83] facilitate access **• Providing services after hours** [91] **• Welcoming and safe spaces** [55, 91] made services more inviting		**• Location of services** [55], transport including proximity to public transport [61, 87], opening hours [79] and inability to pre-book appointments [55] **•** A lack of separate entrances and waiting rooms for men and women meant **facilities were not culturally appropriate**, and **privacy of waiting rooms** was also an issue [55]	**• Location of services** [79], transport including proximity to public transport [79], visibility of service [79] and opening hours [79]
	**Equity and non-discrimination**	**• Low or no cost services** [83, 91] facilitate access **• Adolescents support other adolescents** by providing advice and reassurance to reduce shame in relation to seeking help [87]		**• Cost of services** [70, 91] and **adolescents’ personal circumstances** [61] often prevent or limited service access **• Embarrassment, shame, or fear** [55, 66, 91] often prevent adolescents from seeking and accessing services	**• Embarrassment, shame, or fear** [79] often prevent adolescents from seeking and accessing services
	**Cultural safety**	**•** Adolescents sought out **Indigenous specific services** [61, 83] or services that provided **culturally safe environments and care** [55, 61, 83, 87] **• Cultural protocols** were observed with providers and adolescents being the same gender [55] **•** The presence of **Indigenous health care providers and their role in the provision of culturally safe care** [61, 87]		**•** Many **providers had inadequate training: cultural** [70]	
**Demand**	**Adolescent health literacy**	**• Increasing health literacy** [91] **• Adolescents’ willingness to seek health care** [61, 87] facilitated access to care		**• An assumed or lower level of health literacy** [55, 61, 91], communication skills [55] and attitude of adolescents [55] inhibited access to care **• Adolescents’ ability to make and maintain appointments** [61, 87]	**• A lower level of health literacy** [79] and health seeking behaviour [65] were barries to adolescents seeking care and navigating the health care system
	**Community support**	**• Parent/family and community engagement and involvement in care** [62, 71]	**• Parent/family and community engagement and involvement in care** [62]	**• A lack of community and family acceptance or support** [55, 62] hindered adolescents’ access to care	**• A lack of community and family acceptance or support** [62] hindered adolescents’ access to care
	**Data and quality improvement**				
	**Adolescents’ participation**				**• Disempowerment** especially among females and LGBTQI adolescents [66]

Among health care providers and parents/guardians/community members only two enablers were identified: availability of providers (Appropriate Package of Services) [57], and parent/family and community engagement and involvement in care (Community Support) [62]; and seven barriers across seven of the nine standards: workforce challenges (Appropriate Package of Services) [65, 66], a lack of provider privacy, confidentiality and trust (Providers’ Competencies) [62, 66, 79], location of services (Facility Characteristics) [79], embarrassment, shame, or fear (Equity and Non-discrimination) [79], a lower level of health literacy [79] and health seeking behavior [65] (Adolescent health literacy), a lack of community and family acceptance or support (Community Support) [62], and disempowerment (Adolescents’ participation) [66].

### Urban status

Overall, there were more studies conducted in non-urban settings and therefore more enablers and barriers identified by both adolescents and health care providers and parents/guardians/community members in non-urban settings compared to urban settings (Table 6). Furthermore, there were similarities and differences in the enablers and barriers identified relating to eight of the nine standards across the two settings. Notable similarities identified as enablers were associated with Provider Competencies—provider trust, respect, and relationships with adolescents and families [52, 55, 66, 69, 83, 88, 91], providers who maintain privacy and confidentiality [52, 83, 88, 91] and positive provider qualities [52, 69, 79, 83]; Equity and Non-discrimination – low or no cost services [69, 83, 90, 91]; Cultural safety – culturally safe environments and care [52, 55, 61, 69, 72, 83, 87], and the Indigenous workforce and their role in the provision of culturally safe care [52, 61, 69, 85, 87]; and barriers: Appropriate Package of Services – services not available when required [55, 68, 70, 85, 87]; Cultural Safety – many providers did not have adequate training about Indigenous culture [65, 70]; and Community Support – fear of child welfare services and police involvement if adolescents and families accessed services [68, 69, 85]. Notable differences in enablers and barriers were identified under eight of the nine standards, particularly relating to Providers’ Competencies, Facility Characteristics, Cultural Safety, Adolescent Health Literacy, Community Support, and Adolescent Participation.

**Table 6:** Enablers and barriers associated with Indigenous adolescents accessing primary health care services, by study setting^a^

		**Enablers**		**Barriers**	
		**Urban**	**Non-urban**	**Urban**	**Non-urban**
**Supply**	**Appropriate package of services**	**• Care that meets the needs of adolescents** [83] **• Provision of additional services:** telehealth [90] and transport [83] **• Flexible approach to care and pathways** [70]	**• Adolescent appropriate care** that centres adolescents, families and community [69] and is designed for adolescents [52, 72, 88] **• Provision of additional services**: outreach [52, 55], interpreter [52], transport [61, 87], and social support such as ‘Women’s nights’, and health hardware like condoms [87] **• Flexible approach to care** [88] **• Engagement staff** to engage with and follow up adolescents [88] **• Availability of gender-matched health care providers** [52] **•** Technology and **adolescent specific tools/assessments** assist with the health and wellbeing assessment of adolescents [72]	**• Services not available when required** [70, 85] **• Not receiving care that is needed** [90] **•** The **disconnect between health services** [70] impacted continuity of care **• Limited access to health hardware** – condoms and other forms of contraception [70] **• Workforce challenges**: appropriately aged and gendered providers [83]	**• Services not available when required** [55, 68, 87], including specialist services [59], outreach [68, 69], and transport [52] **• Wait times** [52, 88] **• A lack of follow up and recall** makes it difficult for staff to provide care [88] **• Limited access to health hardware** – condoms and other forms of contraception [61, 87] **• A lack of staffing and resources** [88], including within the community prevented the provision of services and care needed, e.g., emergency housing, in-community alcohol and drug rehabilitation services, psychotherapy, and financial assistance [68] **• Workforce challenges**: transient nature of the providers in remote communities [52, 66]
	**Providers’ competencies**	**• Providers who built trust, respect, and relationships with adolescents and families** [83] **• Providers who maintain privacy and confidentiality** [83] and were honest and non-judgmental [83] were seen to have **positive provider qualities**	**•** Providers who engaged in the community [68] and **built trust, respect, and relationships with adolescents and families** [52, 55, 66, 69, 88] **• Providers who maintain privacy and confidentiality** [52, 88] and were open, respectful, patience, non-judgemental, had a wholistic approach, an open door policy [88], were flexible [52, 69, 79], and went above and beyond their role [88] were seen to have **positive provider qualities**, which provided a **positive experience** [61, 87] **• Provides who listen and had good communication skills** [52, 88]		**• A lack of provider privacy, confidentiality, and trust** [52, 66, 79] **• Previous negative experience with providers** [61, 68, 69, 87] **• Providers reluctant to engage with adolescents and their families** [68] **• Provider isolation** is a challenge for those working in remote communities [69]
	**Facility characteristics**	**• Services with same day appointments, walk-ins** [83]	**• Location of services** [68] **• Welcoming and safe space** [55, 68, 69] **• Being able to book appointments** [52]		**• Location of services** [55, 68, 79], transport including proximity to public transport [61, 79, 87], opening hours [79], and inability to pre-book appointments [55] **•** A lack of separate entrances and waiting rooms for men and women meant **facilities were not culturally appropriate**, and **privacy of waiting rooms** was also an issue [52, 55, 88]
	**Equity and non-discrimination**	**• Low or no cost services** [83, 90]	**• Low or no cost services** [69]	**• Cost of services** [70]	**• Cost of services** [79] and **adolescents personal circumstances** [61] **• Experience of embarrassment, shame, or fear** [52, 55, 61, 66, 68, 69, 79, 80, 87]
	**Cultural safety**	**•** Adolescents sought out **Indigenous specific services** [83] or services that provided **culturally safe environments and care**, which reflected adolescents’ culture and beliefs [83] **•** The presence of **Indigenous workforce and their role in the provision of culturally safe care** [85]	**• Culturally safe environments and care** [52, 55, 61, 69, 72, 87] **• Cultural protocols were observed** with providers and adolescents being the same gender [52] **• Indigenous workforce and their role in the provision of culturally safe care** [52, 61, 69, 87]	**•** Many **providers had inadequate training:** cultural [70]	**• Colonisation and intergenerational trauma** impacts adolescents and their family’s ability to engage with and access care [68] **• No Integration or disconnect between traditional & cultural practices** [69, 80] **• Absence of cultural protocols**: restrictions around family relationships, skin groups and gender [69] and lack of a separate entrance and waiting room for men and women [55] inhibits access **• A lack of Indigenous health care providers** [52, 69] and the challenges they face working and being from the community [68] **• Absence of culture and language** [69] prevented families from understanding care **•** Many **providers had inadequate training:** limited or no understanding of two-spirit and gender and sexuality in the Indigenous context [65]
**Demand**	**Adolescent health literacy**	**• Health literacy**: awareness of services [56]	**• Adolescents’ willingness to seek health care** [61, 87] **• Adolescents support other adolescents** by providing advice and reassurance to reduce shame in relation to seeking help [87] **• Delivery of health promotion in adolescent settings**, e.g., in schools, and incentives [88]		**• A lower level of health literacy** [52, 55, 61, 68, 69, 79, 88] and health seeking behaviours – attitude [55] and perceived need for help [69] or prioritising their own health [52] were barriers to adolescents seeking care and navigating the health care system **• Adolescents’ ability to maintain appointments** [61, 87]
	**Community support**		**• Family and community engagement** [68, 69]	**• A lack of guidance on how to communicate with adolescents** about safe sex, mental health, and suicide [89] **• Fear of child welfare services and police involvement** [85]	**• A lack of family engagement and support** [55] and involvement in treatment decisions [69] **• Family issues**: competing priorities of parents [69] **• Fear of child welfare services and police involvement** [68, 69]
	**Data and quality improvement**				
	**Adolescents’ participation**		**• Adolescent engagement beyond health care**: cultural and other activities [65]		**• Disempowerment** especially among females and LGBTQI adolescents [66]

^a^Table includes results from studies which could be characterized by study setting (urban or non-urban)

## Recommendations

Based on evidence from this review and the Global standards [6] we have developed a set of recommendations for policy and service delivery reform. These recommendations focus on addressing the enablers and barriers associated with each of the nine standards and aim to improve the accessibility of primary health care services for Indigenous adolescents (Table 7).

## Discussion

This is the first review to synthesize findings exploring enablers and barriers to primary health care for Indigenous adolescents. The review synthesized literature from 41 studies that identified enablers and barriers to primary health care for Indigenous adolescents. Across the included studies, more barriers were identified than enablers, and against the WHO Global standards most enablers and barriers related to supply factors. The review identified Indigenous adolescents experience similar enablers and barriers to primary health care as non-Indigenous adolescents [8–10], however, there are additional enablers and barriers related to cultural safety that Indigenous adolescents also experience.

Previous studies have identified numerous barriers to primary health care among adolescents, such as availability of services, cost, location of services, inconvenient opening hours, adolescents lack awareness of services, lack of confidentiality, lack of provider knowledge and skills, and absence of legal frameworks [8–10, 18, 92–99]. While these have also been identified by our review, it’s possible that when these barriers are experienced by Indigenous adolescents they are exacerbated by complex historical factors, intergenerational trauma, racism, and socioeconomic determinants of health [20, 36–38], and more complex health needs [21]. Furthermore, enablers and barriers identified under cultural safety were significant to Indigenous adolescents accessing primary health care services. Cultural safety included enablers and barriers related to culturally appropriate services, culturally safe environment and care, traditional and cultural practices, cultural protocols, Indigenous health care providers, cultural training for health care providers, and colonization, intergenerational trauma, and racism. The absence of these factors or the presence of them in the case of colonization, intergenerational trauma and racism, have been previously identified as barriers to heath care access and having a negative effect on the health and wellbeing of Indigenous people [100–102]. Importantly, the provision of culturally safe spaces and care, particularly the presence of Indigenous health care workers and their role in facilitating culturally safe care, were significant enablers to Indigenous people accessing primary health care services [103–105].

It is well documented Indigenous adolescents experience an excess burden of preventable and treatable diseases. Indigenous adolescents continue to experience communicable diseases typical of childhood [21, 106]. The prevalence of injury, violence, and mental health is highest among Indigenous adolescents compared to non-Indigenous adolescents and are prevailing causes of premature mortality [20, 107]. The early on-set of chronic diseases such as obesity, diabetes and cardiovascular disease are more prevalent in Indigenous adolescents than non-Indigenous adolescents [20, 21, 106, 108]. This excess burden requires a different approach, knowledge, and skills to address, as well as additional care and resources to mitigate any long-term impact.

There are numerous examples available which demonstrates what works in relation to addressing Indigenous health and wellbeing, and adolescent health. Indigenous health services have a proven track record of improving the health of Indigenous people [109]. Their scope of service delivery is far more than comprehensive, it is holistic and robust and tailored to meet the health and wellbeing needs of community and guided by Indigenous leadership and community-control which is critical for ensuring Indigenous representation in decision-making [29, 110]. Furthermore, Indigenous health services are culturally safe, often situated within Indigenous communities, and seen as beacons of Indigenous excellence that facilitate self-determination [29, 110, 111]. Health care providers are instrumental in the delivery of care [104], as this review has identified they can act as an enablers or barriers to care. The WHO has recognized the unique role health care providers play in providing care to adolescents and has developed the *Core competencies in adolescent health and development for primary health care providers* [112]. Similarly, Indigenous health care providers are an important component to the delivery of culturally safe care, due to their shared Indigenous identity, lived experience and often belonging to the communities in which they practice [103, 113–115]. More broadly, evidence has identified models of care that center and involve adolescents in decision making work. For example, *headspace* a national youth mental healthcare service in Australia was designed in recognition to the unmet need and the high incidence and prevalence of mental health issues among young people [116]. Similar models of care such as school-based health clinics in the USA have demonstrated that the provision of care within a youth setting such as a school can increase access to care, health and educational outcomes [117–119].

The review highlighted several gaps in the evidence, including limited evidence on Indigenous adolescents’ access to primary health care services, as well as the perspectives of Indigenous male adolescents; younger adolescents; urban settings; and limited breadth and depth of evidence across primary health care services and health conditions. Furthermore, studies among Indigenous adolescents accessing primary health care services in urban settings is limited and further research is required, including understanding the enablers and barriers to care on both the supply and demand side, and how primary health care services can be improved to better meet the health and wellbeing needs of Indigenous adolescents. Similarly, there was a paucity of studies that focused exclusively on Indigenous male adolescents within primary health care services. The lack of evidence and health research among Indigenous males including Indigenous male adolescents in primary health care services requires action, especially considering Indigenous adolescent males are at greater risk of experiencing mortality and morbidity, their experience of social and cultural determinants of health, and are less likely to engage with primary health care services than female Indigenous adolescents [20, 21, 120].

Our review had several limitations. The search strategy and selection criteria excluded studies on prevention and screening, which are core components of comprehensive primary health care; however, the reviewed focused on the responsive (acute) care component of primary health care services. We defined adolescents as those aged 10–24 years, as such we may have missed studies which did not specify age and referred to younger adolescents as children and older adolescents as adults. Similarly, while the review included 41 studies, there is always a possibility that the review did not identify all potential articles. Most studies were single health service studies, rarely did studies show the depth of adolescent patterns of care across multiple health care services or regions, and most focused predominately on specific diseases or similar health conditions. Furthermore, there were very few quantitative studies that showed the relationship between the barriers & enablers of primary health care, access and health outcomes. Overall, most studies scored highly against the Joanna Briggs Institute tools, however, not against the Aboriginal and Torres Strait Islander Quality Appraisal Tool, where overall quality and reporting for each criterion was relatively poor. Studies were assessed using the information reported, however, this does not mean the processes and activities for which the Aboriginal and Torres Strait Islander Quality Appraisal Tool assess against did not occur. Even though reporting guidelines for Indigenous health research now exist [121], journals do not require studies to demonstrate how they meet the guidelines. The lack of reporting using Indigenous guidelines is of concern given the number of national and international research guidelines [122–125] that call for Indigenous research to reflect Indigenous ways of knowing, being and doing; including a greater push towards Indigenous research being conducting with and by Indigenous researchers.

If we want primary health care services that are more accessible to adolescents, then these services need to consider Indigenous adolescents, their health needs [21] and the barriers and enablers they experience when accessing services. Based on this review and the WHO Global standards for quality health-care services for adolescents, we have developed nine recommendations for policy and service delivery reform that focus on the enablers that improve the accessibility of primary health care services for Indigenous adolescents (Table 7). However, the full implementation of the recommendations will require a commitment by government at all levels to fully prioritize Indigenous adolescent health programs and policy, with an accompanying commitment to resourcing, staffing and programs focused on Indigenous adolescents.

**Table 7:** Recommendations for improving the accessibility of primary health care services for Indigenous adolescents

1. Health care services and providers ensure privacy and confidentiality which enables them to build trust, respect, and relationships with Indigenous adolescents and their families.
2. Facilities, health care services and providers acknowledge and implement culture, cultural protocols and practices that foster a culturally safe environment and care for Indigenous adolescents.
3. The capacity and competencies of health care providers is strengthened in adolescent and Indigenous health including cultural training.
4. Build the Indigenous health workforce and their capacity to delivery adolescent health care within and for their community.
5. Health literacy of Indigenous adolescents, their family and community are strengthened to enable adolescents to make informed decisions about their health and wellbeing including being involved in decisions regarding their own care, and to assist with navigating the health care system.
6. Health care services provide a package of services which meet the complex health and wellbeing needs of Indigenous adolescents, including access to adolescent specialists and outreach services.
7. Health care services are made more accessible to Indigenous adolescents by locating services in settings which are move convenient to adolescents, close to public transport or transport is provided by services, and services have extended operating hours and are free to access.
8. Indigenous adolescents are involved in the planning and designed of health care services that center Indigenous adolescents, their families, and communities.
9. Health care services are made more equitable and accessible to Indigenous adolescents and services and providers provide services without discrimination.

## Conclusion

Indigenous adolescents experience the same barriers and enablers to accessing primary health care services as adolescent more broadly, however, these may be exacerbated by complex historical and socioeconomic factors. Indigenous adolescents also experience additional enablers and barriers to accessing primary health care services. These enablers and barriers were related to aspects of cultural safety – culturally appropriate services, environment, and care, and were found to be significant to Indigenous adolescents’ experience of accessing primary health care services. This review provides important evidence to inform how services, organizations and governments create adolescent accessible primary health care services that specifically meet the needs of Indigenous adolescents.

### Supplementary Information

**Additional file 1.**

## Data Availability

Data extracted from included studies for this review are available from the corresponding author on reasonable request.

## Abbreviations

USA: United States of America
WHO: World Health Organization
