# Supplementary material for: Enablers and barriers to primary health care access for Indigenous adolescents: a systematic review and meta-aggregation of studies across Australia, Canada, New Zealand, and USA
Source: BMC Health Serv Res. 2024 Apr 30;24:553. doi: 10.1186/s12913-024-10796-5 (PMC11062015; doi:10.1186/s12913-024-10796-5)
Supplement: Supplementary file 1 — Additional file 1. [file 12913_2024_10796_MOESM1_ESM.docx]

**Supplementary material**

Table of Contents

[**Table S1: Search strategy*** 2](#_Toc126333616)

[**Table S2: Quality Assessment – Joanna Briggs Institute tools** 4](#_Toc126333617)

[**Table S3: The Aboriginal and Torres Strait Islander Quality Appraisal Tool** 6](#_Toc126333618)

## **Table S1: Search strategy***

|  | **Indigenous** | **Country** | **Adolescent** | **Primary health care** |
| --- | --- | --- | --- | --- |
| **Subject Headings**  **[MeSH Terms]** | “Australian aborigines”  “American native continental ancestry group”  “American, native”  “American Indian”  “American Indian, north”  “Inuit”  “Oceanic ancestry group” | “Australia”  “New Zealand”  “Canada”  “United States of America”  “Hawaii”  “Alaska” | “Adolescence”  “Adolescent”  “Teen”  “Teenager”  “Youth”  “Adult, young”  “Young adults”  “Minors | “primary health care”  “primary healthcare”  “primary care”  “primary care nursing”  “comprehensive health care”  “family practice”  “general practice”  “ambulatory care”  “ambulatory care facilities”  “community health care”  “community health centre”  “community health centres”  “community health service”  “community health services”  “community mental health centres”  “community mental health services”  “counseling”  “family planning”  “family planning centre”  “family planning service”  “family planning services”  “home health care agencies”  “maternal health service”  “maternal health services”  “community mental health centres”  “indigenous health service”  “adolescent health service”  “student health service” |
| **Free text terms**  **(searched in title & abstract [tiab]** | (Aborigin  Indigenous people  Indigenous Australia*  Maori*  First nation*  First people  Torres Strait*  Inuit*  Metis*  Yupik*  Aleut*  Inupiat*  Eskimo  American Indian  Native American  Alaskan native  native hawaii*  native people*  trib* people*  native population  trib* population* | Australia*  New Zealand*  Aotearoa*  Canada*  Hawaii*  Alaska*  United States of America  United States  North America* | Adolescen*  Teen*  Minors  Young adult*  Early adult*  Emerging adult*  Youth  Juvenile Twenties  10 year old*  11 year old*  12 year old*  13 year old*  14 year old*  15 year old*  16 year old*  17 year old*  18 year old*  19 year old*  20 year old*  21 year old*  22 year old*  23 year old*  24 year old* | primary health care  primary healthcare  comprehensive healthcare  comprehensive health care  community health care  community controlled health care  community healthcare  maternal-child health services  community health nursing  community mental health services  maternal health services  community mental health service  community mental health care  mobile health units  reproductive health services  family planning service  sexual health service  sexual health centre  general practice  family medicine  family practice  perinatal health care  perinatal health service  prenatal care  prenatal service  antenatal service  antenatal care  pregnancy care  pregnancy service  drug and alcohol service  drug and alcohol care  adolescent health care  adolescent health service  student health care  student health service  child health service  aboriginal health service  indigenous health service |

*Limits: Studies published in English from Jan 1 2002 to July 26 2021

## **Table S2: Quality Assessment – Joanna Briggs Institute tools**

|  | Congruity between philosophical perspective and research methodology | Congruity between research methodology and research question or objectives | Congruity between research methodology and methods used to collect data | Congruity between research methodology and representation and analysis of data | There is congruence between research methodology and interpretation of results | A statement locating the researcher culturally or theoretically | Influence of the researcher on the research, and vice-versa, is addressed | Representation of participants and their voices | Ethical approval by an appropriate body | Relationship of conclusions to analysis, or interpretation of the data | Criteria for inclusion in the sample clearly defined | Study subjects and the setting described | The exposure measured in a valid and reliable way | Objective, standard criteria used for measurement of the condition | Confounding factors identified | Strategies to deal with confounding factors stated | Outcomes measured in a valid and reliable way | Appropriate statistical analysis used |
| --- | --- | --- | --- | --- | --- | --- | --- | --- | --- | --- | --- | --- | --- | --- | --- | --- | --- | --- |
| Ameratunga et al (2019) | N/A | N/A | N/A | N/A | N/A | N/A | N/A | N/A | N/A | N/A | Y | Y | Y | Y | Y | Y | Y | Y |
| Angelino et al (2020) | Y | Y | Y | Y | Y | N | N | Y | Y | Y | N/A | N/A | N/A | N/A | N/A | N/A | N/A | N/A |
| Auger (2019) | Y | Y | Y | Y | Y | N | U | Y | Y | Y | N/A | N/A | N/A | N/A | N/A | N/A | N/A | N/A |
| Bell et al (2020) | Y | Y | Y | Y | Y | Y | U | Y | Y | Y | N/A | N/A | N/A | N/A | N/A | N/A | N/A | N/A |
| Canuto et al (2018) | Y | Y | Y | Y | Y | N | Y | Y | N | Y | N/A | N/A | N/A | N/A | N/A | N/A | N/A | N/A |
| Corosky et al (2016) | Y | Y | Y | Y | Y | N | N | Y | Y | Y | N/A | N/A | N/A | N/A | N/A | N/A | N/A | N/A |
| Dickerson et al (2011) | Y | Y | Y | Y | Y | N | N | Y | Y | Y | N/A | N/A | N/A | N/A | N/A | N/A | N/A | N/A |
| Dowsett et al (2019) | Y | Y | Y | Y | Y | N | N | Y | Y | Y | N/A | N/A | N/A | N/A | N/A | N/A | N/A | N/A |
| Etter et al (2019) | Y | Y | Y | Y | Y | N | N | Y | Y | Y | N/A | N/A | N/A | N/A | N/A | N/A | N/A | N/A |
| Fraser et al (2021) | Y | Y | Y | Y | Y | N | N | Y | Y | Y | N/A | N/A | N/A | N/A | N/A | N/A | N/A | N/A |
| Freedenthal et al (2007) | Y | Y | Y | Y | Y | N | Y | Y | Y | Y | N/A | N/A | N/A | N/A | N/A | N/A | N/A | N/A |
| Garrett et al (2022) | Y | Y | Y | Y | Y | N | N | Y | Y | Y | N/A | N/A | N/A | N/A | N/A | N/A | N/A | N/A |
| Greenstein et al (2016) | Y | Y | Y | Y | Y | N | N | Y | Y | Y | N/A | N/A | N/A | N/A | N/A | N/A | N/A | N/A |
| Hendrickx et al (2020) | Y | Y | Y | Y | Y | N | N | Y | Y | Y | N/A | N/A | N/A | N/A | N/A | N/A | N/A | N/A |
| Hummel et al (2022) | Y | Y | Y | Y | Y | N | N | Y | Y | Y | N/A | N/A | N/A | N/A | N/A | N/A | N/A | N/A |
| Hutt-MacLeod et al (2019) | Y | Y | Y | Y | Y | N | N | Y | Y | Y | N/A | N/A | N/A | N/A | N/A | N/A | N/A | N/A |
| Johnston et al (2015) | Y | Y | Y | Y | Y | N | N | Y | Y | Y | N/A | N/A | N/A | N/A | N/A | N/A | N/A | N/A |
| Kalucy et al (2019) | Y | Y | Y | Y | Y | N | N | Y | Y | Y | N/A | N/A | N/A | N/A | N/A | N/A | N/A | N/A |
| Kurtin et al (2009) | Y | Y | Y | Y | Y | N | N | Y | N | Y | N/A | N/A | N/A | N/A | N/A | N/A | N/A | N/A |
| Lau et al (2012) | N/A | N/A | N/A | N/A | N/A | N/A | N/A | N/A | N/A | N/A | Y | Y | Y | Y | Y | Y | Y | Y |
| Lawton et al (2016) | Y | Y | Y | Y | Y | Y | N | Y | Y | Y | N/A | N/A | N/A | N/A | N/A | N/A | N/A | N/A |
| Makowharemahihi et al (2014) | Y | Y | Y | Y | Y | N | N | Y | Y | Y | N/A | N/A | N/A | N/A | N/A | N/A | N/A | N/A |
| Martel et al (2020) | Y | Y | Y | Y | Y | N | U | Y | Y | Y | N/A | N/A | N/A | N/A | N/A | N/A | N/A | N/A |
| McClintock et al (2013) | Y | Y | Y | Y | Y | N | U | Y | Y | Y | N/A | N/A | N/A | N/A | N/A | N/A | N/A | N/A |
| McClintock et al (2016) | N/A | N/A | N/A | N/A | N/A | N/A | N/A | N/A | N/A | N/A | Y | Y | Y | Y | N | N | Y | Y |
| Mooney-Somers et al (2009) | Y | Y | Y | Y | Y | N | N | Y | Y | Y | N/A | N/A | N/A | N/A | N/A | N/A | N/A | N/A |
| Mooney-Somers et al (2009) | Y | Y | Y | Y | Y | N | N | Y | Y | Y | N/A | N/A | N/A | N/A | N/A | N/A | N/A | N/A |
| Reibel et al (2015) | Y | Y | Y | Y | Y | N | N | Y | Y | Y | N/A | N/A | N/A | N/A | N/A | N/A | N/A | N/A |
| Robards et al (2019) | Y | Y | Y | Y | Y | N | N | Y | Y | Y | N/A | N/A | N/A | N/A | N/A | N/A | N/A | N/A |
| Rose et al (2021) | Y | Y | Y | Y | Y | N | N | Y | Y | Y | N/A | N/A | N/A | N/A | N/A | N/A | N/A | N/A |
| Sabbioni et al (2018) | Y | Y | Y | Y | Y | N | N | Y | Y | Y | N/A | N/A | N/A | N/A | N/A | N/A | N/A | N/A |
| Saftner et al (2014) | Y | Y | Y | Y | Y | N | N | Y | Y | Y | N/A | N/A | N/A | N/A | N/A | N/A | N/A | N/A |
| Salvador et al (2016) | Y | Y | Y | Y | Y | N | Y | Y | Y | Y | N/A | N/A | N/A | N/A | N/A | N/A | N/A | N/A |
| Santhanam et al (2006) | Y | Y | Y | Y | Y | N | Y | Y | Y | Y | N/A | N/A | N/A | N/A | N/A | N/A | N/A | N/A |
| Schultz et al (2019) | N/A | N/A | N/A | N/A | N/A | N/A | N/A | N/A | N/A | N/A | Y | Y | Y | Y | Y | Y | Y | Y |
| Stewart et al (2013) | Y | Y | Y | Y | Y | N | N | Y | U | U | N/A | N/A | N/A | N/A | N/A | N/A | N/A | N/A |
| Warwick et al (2019) | Y | Y | Y | Y | Y | N | N | Y | Y | Y | N/A | N/A | N/A | N/A | N/A | N/A | N/A | N/A |
| Warwick et al (2021) | Y | Y | Y | Y | Y | Y | N | Y | Y | Y | N/A | N/A | N/A | N/A | N/A | N/A | N/A | N/A |
| Westerman (2010) | Y | U | U | U | U | N | N | N | N | Y | N/A | N/A | N/A | N/A | N/A | N/A | N/A | N/A |
| Williamson et al (2010) | Y | Y | Y | Y | Y | N | N | Y | Y | Y | N/A | N/A | N/A | N/A | N/A | N/A | N/A | N/A |
| Yi et al (2015) | Y | Y | Y | Y | Y | N | N | Y | Y | Y | N/A | N/A | N/A | N/A | N/A | N/A | N/A | N/A |

Key: Y: Yes; U: Unclear; N: No; N/A: Not applicable

## **Table S3: The Aboriginal and Torres Strait Islander Quality Appraisal Tool (modified)**

|  | Did the research respond to a need or priority determined by the community? | Was community consultation and engagement appropriately inclusive? | Did the research have Indigenous research leadership? | Did the research have Indigenous governance? | Were local community protocols respected and followed? | Did the researchers negotiate agreements in regards to rights of access to Indigenous peoples existing intellectual and cultural property? | Did the researchers negotiate agreements to protect Indigenous peoples’ ownership of intellectual and cultural property created through the research? | Did Indigenous peoples and communities have control over the collection and management of research materials? | Was the research guided by an Indigenous research paradigm? | Does the research take a strengths-based approach, acknowledging and moving beyond practices that have harmed Indigenous peoples in the past? | Did the researchers plan and translate the findings into sustainable changes in policy and/or practice? | Did the research benefit the participants and Indigenous communities? | Did the research demonstrate capacity strengthening for Indigenous individuals? | Did everyone involved in the research have opportunities to learn from each other? |
| --- | --- | --- | --- | --- | --- | --- | --- | --- | --- | --- | --- | --- | --- | --- |
| Ameratunga et al (2019) | Y | N | Y | N | N | N | N | N | N | N | N | N | N | N |
| Angelino et al (2020) | Y | Y | Y | U | Y | N | N | N | Y | Y | P | Y | N | N |
| Auger (2019) | Y | Y | Y | N | Y | N | N | Y | Y | Y | P | Y | Y | Y |
| Bell et al (2020) | Y | Y | Y | Y | Y | N | N | Y | Y | Y | Y | Y | Y | Y |
| Canuto et al (2018) | Y | Y | Y | Y | Y | N | Y | Y | Y | Y | N | Y | Y | Y |
| Corosky et al (2016) | Y | Y | N | N | Y | N | N | U | Y | Y | N | Y | N | Y |
| Dickerson et al (2011) | Y | Y | Y | N | Y | N | N | N | U | Y | Y | Y | N | N |
| Dowsett et al (2019) | Y | U | U | Y | N | N | N | N | N | N | N | N | N | N |
| Etter et al (2019) | Y | Y | Y | Y | Y | N | N | Y | Y | Y | Y | Y | Y | Y |
| Fraser et al (2021) | Y | U | U | Y | Y | N | N | N | Y | Y | N | Y | Y | Y |
| Freedenthal et al (2007) | Y | N | N | N | N | N | N | N | N | N | N | N | N | N |
| Garrett et al (2022) | N | N | N | N | N | N | N | N | N | N | N | N | N | N |
| Greenstein et al (2016) | Y | N | N | N | N | N | N | P | N | N | Y | Y | N | N |
| Hendrickx et al (2020) | Y | Y | Y | Y | Y | N | N | N | P | N | Y | Y | N | N |
| Hummel et al (2022) | Y | Y | U | Y | Y | N | N | U | Y | Y | N | P | N | N |
| Hutt-MacLeod et al (2019) | Y | Y | Y | Y | Y | N | N | N | Y | Y | Y | Y | Y | Y |
| Johnston et al (2015) | Y | U | U | N | N | N | N | N | N | N | N | N | N | N |
| Kalucy et al (2019) | Y | Y | Y | Y | Y | Y | Y | Y | Y | Y | Y | Y | N | N |
| Kurtin et al (2009) | Y | N | N | N | N | N | N | N | N | N | N | N | N | N |
| Lau et al (2012) | Y | N | N | N | N | N | N | N | N | NY | N | N | N | N |
| Lawton et al (2016) | Y | N | Y | Y | Y | N | N | N | Y | Y | N | Y | N | Y |
| Makowharemahihi et al (2014) | Y | Y | Y | Y | Y | N | N | N | Y | Y | N | Y | N | N |
| Martel et al (2020) | Y | Y | Y | Y | Y | N | N | N | U | Y | Y | Y | N | N |
| McClintock et al (2013) | Y | Y | Y | Y | Y | N | N | N | Y | Y | P | Y | N | N |
| McClintock et al (2016) | Y | Y | Y | Y | Y | N | N | Y | Y | Y | N | Y | Y | Y |
| Mooney-Somers et al (2009) | Y | Y | Y | Y | Y | N | Y | N | Y | Y | Y | Y | Y | Y |
| Mooney-Somers et al (2009) | Y | Y | Y | Y | Y | N | N | P | Y | N | N | Y | Y | Y |
| Reibel et al (2015) | Y | Y | Y | Y | Y | N | N | N | Y | Y | N | Y | Y | Y |
| Robards et al (2019) | Y | N | N | N | N | N | N | N | N | N | Y | N | N | N |
| Rose et al (2021) | Y | N | N | N | N | N | N | N | N | N | N | N | N | N |
| Sabbioni et al (2018) | Y | N | Y | N | N | N | N | N | Y | Y | N | Y | N | N |
| Saftner et al (2014) | Y | N | Y | N | N | N | N | N | N | N | Y | Y | N | N |
| Salvador et al (2016) | Y | Y | N | N | N | N | N | N | N | Y | N | Y | N | N |
| Santhanam et al (2006) | Y | Y | N | N | Y | N | N | N | N | Y | Y | Y | Y | Y |
| Schultz et al (2019) | Y | Y | Y | Y | Y | N | N | Y | P | Y | P | Y | Y | Y |
| Stewart et al (2013) | Y | Y | Y | Y | Y | Y | Y | Y | P | Y | Y | Y | Y | Y |
| Warwick et al (2019) | Y | Y | U | U | Y | N | N | N | Y | Y | Y | Y | Y | Y |
| Warwick et al (2021) | Y | Y | U | U | Y | N | N | N | Y | Y | Y | Y | Y | Y |
| Westerman (2010) | Y | N | Y | N | N | N | N | N | Y | Y | N | U | N | N |
| Williamson et al (2010) | Y | N | Y | N | N | N | N | N | N | Y | N | Y | N | N |
| Yi et al (2015) | Y | N | N | N | Y | N | N | N | N | Y | N | N | N | N |

Key: Y: Yes; P: Partial; U: Unclear; N: N

## 
